# Crystal Arthropathy in the Setting of Total Knee Arthroplasty

**DOI:** 10.1155/2020/7613627

**Published:** 2020-03-24

**Authors:** Joseph C. Brinkman, Kade S. McQuivey, Justin L. Makovicka, Joshua S. Bingham

**Affiliations:** Department of Orthopedics, Mayo Clinic in Arizona, Phoenix, Arizona, USA

## Abstract

We present a case of an 82-year-old female with a history of right total knee arthroplasty 11 years prior. She was admitted after a ground-level fall and developed progressive pain and swelling of her right knee. She had no history of complications with her total knee replacement. Radiographs of the knee and hip were negative for acute fracture, dislocation, or hardware malalignment. Knee aspiration was performed and revealed inflammatory exudate, synovial fluid consistent with crystal arthropathy, and no bacterial growth. She was diagnosed with an acute gout flare, and her symptoms significantly improved with steroids and anti-inflammatory treatment. Orthopedic surgeons should be aware of the potential for crystal arthropathy in the setting of total joint arthroplasty and evaluate for crystals before treating a presumed periprosthetic joint infection.

## 1. Introduction

Total knee arthroplasty (TKA) is an effective treatment option for improving pain and function in patients with end-stage osteoarthritis [1]. The rate of TKA utilization in the United States has increased substantially in recent years with a projected rate of 3.48 million annual procedures by 2030 [2, 3]. One major complication of TKA is periprosthetic joint infection (PJI), which develops in an estimated 1-2% of primary cases and up to 9% in revision cases [2, 4, 5]. These infections portend a higher mortality and often require burdensome treatment such as additional surgery and long-term antibiotics [6, 7].

Early diagnosis is crucial for optimal PJI management. However, they can be a difficult diagnosis to establish. In order to assist in diagnosis, the Musculoskeletal Infection Society has created standard criteria for which to base the diagnosis of a PJI wherein two positive cultures or the presence of a sinus tract represent major criteria [8, 9]. Minor criteria are indicated by findings including elevated serum C-reactive protein (>10 mg/L), serum erythrocyte sedimentation rate (>30 mm/h), serum D-dimer (>860 ng/mL), and synovial white blood cell count (>3000 cells/*μ*L); positive alpha-defensin; positive leukocyte esterase; elevated polymorphonuclear percentage > 80%; and synovial CRP above 6.9 mg/L [9]. The utility of polymerase chain reaction (PCR) in detecting PJI has also been reported [10]. Additionally, a high index of suspicion for PJI is warranted when symptoms such as pain, redness, and swelling arise in the setting of prior total joint replacement. However, these clinical symptoms can also be seen in other conditions such as rheumatoid arthritis, gonococcal arthritis, gout, and pseudogout [11].

We present a case in which a patient presented with symptoms consistent with a PJI, but was later found to be suffering from an acute gout flare. The patient experienced complete relief with appropriate pharmacological treatment and did not require surgical management. This case demonstrates the importance of evaluating for crystal arthropathy before presuming an infectious etiology for acute knee symptoms in the setting of a TKA.

## 2. Case

**Figure 1:**
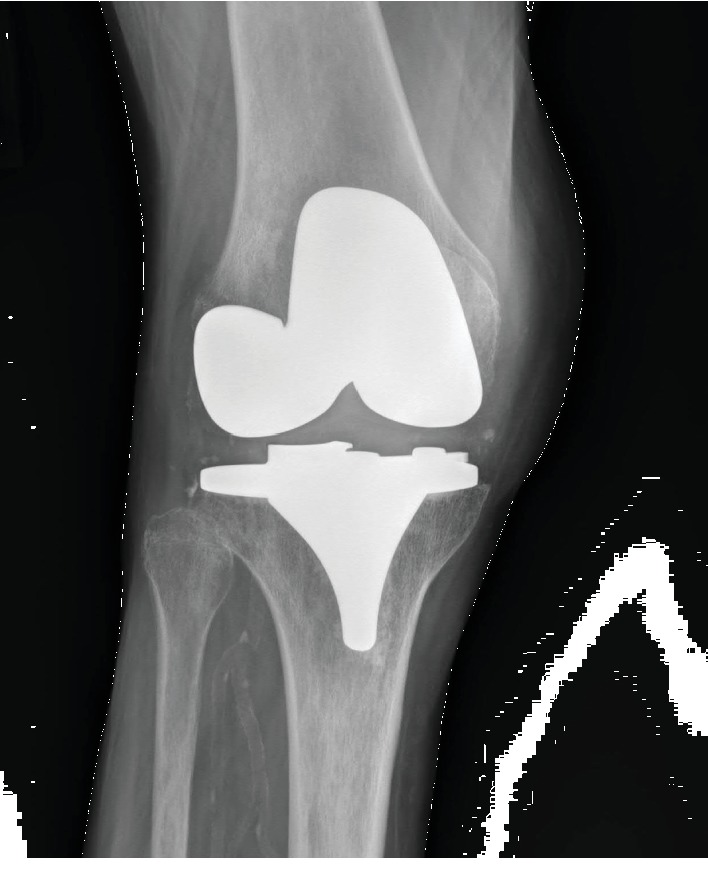
Anteroposterior radiograph of the right knee demonstrating TKA hardware without acute abnormality.

An 82-year old female with a past medical history of alcohol abuse, chronic kidney disease, and right total knee arthroplasty 11 years prior presented to the emergency department after a ground-level fall during a syncopal episode. Her initial complaints included right hip and knee pain, left shoulder pain, and left-sided forehead pain. Radiographs of the left shoulder revealed an acute fracture of the distal clavicle with no additional findings found on radiographs of the right hip, left lower leg, or right knee (Figure 1). Computed tomography (CT) imaging of the head showed a left frontal scalp hematoma without any acute intracranial abnormality; thus, she was admitted for management and workup of her syncopal event.

**Figure 2:**
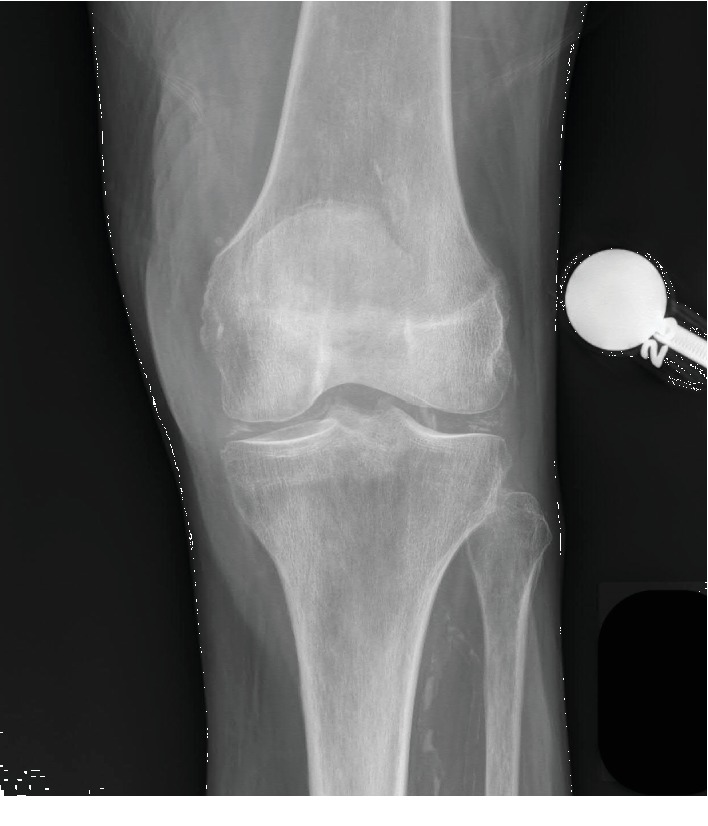
Anteroposterior radiograph of the left knee demonstrating chondrocalcinosis.

On the second day of admission, the patient was noted to have increased tenderness to her left lower leg. A radiograph at this time was negative for acute fracture but demonstrated extensive chondrocalcinosis (Figure 2) of the knee joint. Also, at this time she began complaining of increased pain and swelling to her right knee. The following day, these symptoms continued to worsen and she subsequently underwent further imaging with a right knee CT scan. The scan revealed a large joint effusion without evidence of acute injury. Although she did experience mild intermittent hypertensive and tachycardic episodes, she was afebrile throughout her entire admission and her white blood cell count ranged from 9.5 to 14.4 × 10^9^/L. At this time, the orthopedic surgery service was consulted to assess her right knee symptoms and evaluate for a PJI.

Upon evaluation by the orthopedic service, the patient complained of severe right knee pain that worsened with flexion and extension. Prior to this, she denied any problems with her knee replacement or any history of gout. Physical exam of the right knee was significant for a marked effusion, diffuse tenderness to palpation, and restricted range of motion with guarding. Further workup included erythrocyte sedimentation rate (ESR) and C-reactive protein (CRP) levels, which were both found to be elevated at 91 mm/hr and 213 mg/L, respectively. Given the patient's physical exam findings and elevated ESR/CRP, an arthrocentesis with synovial fluid analysis was performed to evaluate for a PJI. Aspiration yielded 100 mL of serosanguinous fluid, and synovial fluid analysis demonstrated a cell count of 20,833 with 92% neutrophil count. No organisms were seen on gram stain. Synovial fluid crystal analysis was positive for monosodium urate crystals and pyrophosphate crystals.

In light of the aspiration results and her clinical presentation, she was diagnosed with acute gouty arthropathy which was supported by an elevated serum uric acid level of 8.2 mg/dL. Initial management included prednisone, colchicine, and allopurinol. In addition to the therapeutic effect of the aspiration, this regimen offered the patient near full relief of her pain and restoration of her range of motion two days later, at which point she was discharged from the hospital in stable condition. Final cultures were negative, and she remained asymptomatic through four months of follow-up.

## 3. Discussion

Crystal arthropathy due to either gout or pseudogout is rare in the setting of previous total joint arthroplasty. A review of the current literature reveals only 13 reported cases of pseudogout in prosthetic joints [12–21]. Although the majority of these cases were managed pharmacologically, 23% underwent surgical treatment consisting of washout alone or in addition to polyethylene exchange [22]. Periprosthetic gout has also been described in the literature with eight aseptic cases reported [19, 23–29]. Although it has been established that aseptic gouty arthritis can be successfully managed with gout medications alone, this is not the routine treatment. Management of noninfectious cases have involved synovectomy, surgical washout, polyethylene exchange, pharmacological treatment, or a combination of these methods depending on when the infection was recognized [19, 23, 27]. A recent systematic review found that in a cohort in which only 13% of periprosthetic gout cases were proven to have concomitant infection, 59% underwent surgical washout, revision, or polyethylene exchange [22]. As more cases required surgical management than were infected, this review suggests that a significant cohort of patients undergo unnecessary revision or washout. These procedures could potentially be avoided with accurate diagnosis of crystal arthropathy, and surgical management could be reserved for cases refractory to medical management or with definitive signs of infection.

Distinguishing between crystal arthropathy and infection in a prosthetic knee presents a diagnostic challenge. The conditions share a clinical presentation of pain, swelling, erythema, and restricted motion. It has been demonstrated that laboratory studies revealing elevated synovial white blood cells along with elevated erythrocyte sedimentation rate and C-reactive protein indicate infection in prosthetic joints with reliable sensitivity and specificity [30]. These same findings are commonly seen in acute crystal arthropathy, as illustrated in the presented case. Further, in regard to the serum markers, it has been demonstrated that the degree of elevation does not differ significantly between the two entities in the setting of a prosthetic knee [22]. The MSIS criteria are commonly used to diagnose periprosthetic infections, but they do not allow for differentiation of crystal arthropathy. According to these criteria, the synovial cell count and acute inflammatory marker elevation of the presented case indicated acute infection. Analyzing the synovial fluid for crystals was the only means by which crystal arthropathy was indicated, and it subsequently prevented the patient from undergoing an unnecessary surgical washout.

It is critically important to evaluate for and treat PJI when present. However, it is also important to consider the possibility that crystal arthropathy may be responsible for symptoms representing infection in the setting of a total joint arthroplasty. This should be particularly considered in patients with chronic kidney disease, such as the presented case, given the association shared between renal disease and gout [31]. As the majority of crystal arthropathy cases can be successfully managed with medications alone, evaluating for synovial crystals in the workup of a painful prosthetic knee can potentially save the patient from unnecessary surgery, antibiotics, and the sequalae associated with these treatments. If there is a high suspicion for an underlying crystal arthropathy, and the patient is stable, we feel that it is reasonable to start with a trial of medical treatment of the arthropathy before going straight to a potentially unnecessary surgery or more expensive laboratory tests.
